# Expression Levels of *LCORL* Are Associated with Body Size in Horses

**DOI:** 10.1371/journal.pone.0056497

**Published:** 2013-02-13

**Authors:** Julia Metzger, Rahel Schrimpf, Ute Philipp, Ottmar Distl

**Affiliations:** Institute for Animal Breeding and Genetics, University of Veterinary Medicine Hannover, Hannover, Germany; University of Tasmania, Australia

## Abstract

Body size is an important characteristic for horses of various breeds and essential for the classification of ponies concerning the limit value of 148 cm (58.27 inches) height at the withers. Genome-wide association analyses revealed the highest associated quantitative trait locus for height at the withers on horse chromosome (ECA) 3 upstream of the candidate gene *LCORL*. Using 214 Hanoverian horses genotyped on the Illumina equine SNP50 BeadChip and 42 different horse breeds across all size ranges, we confirmed the highly associated single nucleotide polymorphism BIEC2-808543 (−log_10_P = 8.3) and the adjacent gene *LCORL* as the most promising candidate for body size. We investigated the relative expression levels of *LCORL* and its two neighbouring genes *NCAPG* and *DCAF16* using quantitative real-time PCR (RT-qPCR). We could demonstrate a significant association of the relative *LCORL* expression levels with the size of the horses and the BIEC2-808543 genotypes within and across horse breeds. In heterozygous C/T-horses expression levels of *LCORL* were significantly decreased by 40% and in homozygous C/C-horses by 56% relative to the smaller T/T-horses. Bioinformatic analyses indicated that this SNP T>C mutation is disrupting a putative binding site of the transcription factor *TFIID* which is important for the transcription process of genes involved in skeletal bone development. Thus, our findings suggest that expression levels of *LCORL* play a key role for body size within and across horse breeds and regulation of the expression of *LCORL* is associated with genetic variants of BIEC2-808543. This is the first functional study for a body size regulating polymorphism in horses and a further step to unravel the mechanisms for understanding the genetic regulation of body size in horses.

## Introduction

Body size is an important model trait for studying genetic influences on quantitative traits and has been intensely investigated in human and also in domestic animals [1]–[17]. In human, adult height is described to be a complex trait influenced by many genes and environmental factors [5]. Several genetic variants affecting the adult height have been identified using association analyses [5], [6].

In horses, body size is an important criterion for the evaluation of different breeds concerning appearance and function and is crucial for the classification of horses. According to the Fédération Equestre Internationale (FEI) veterinary regulations ponies taking part in any FEI competition have to be measured at the highest point of the withers. The limit height is in accordance with the definition of a pony 148 cm (centimetres) (58.27 inches) without shoes or with competition shoes 149 cm (58.66 inches). If this height is exceeded the animal is then classified as a horse [7]. In some breeds, the limit values are even lower. The American Miniature Horse Association requires a limit height at the last hairs of the mane of 86.4 cm (34 inches). For breeders, body size of horses is an essential parameter to improve marketability, function and performance. The important effect of size for competitive jumping ability in ponies was suggested [8]. Generally, larger animals within each height class possess competitive advantage and performances are evaluated correspondingly [13], [14]. Due to the selection for specific functions, the domestic horse has been modified within breeds into diverse skeletal morphologic types. The heritability of height at the withers was estimated to be medium to high in pony breeds. Particularly, in Haflinger and Shetland ponies high heritabilities at 0.79–0.89 were found [8]–[10] while Icelandic and Hanoverian warmblood showed medium values at 0.5–0.6 [11], [12]. The first attempt to identify patterns of skeletal size and shape variation among domestic horses has been made by principal component analyses [15]. Overall body size was used as a principal component including thirty measurements all over horse's body like head length, height at withers, height at croup, chest width and neck length. It grouped small ponies together with low scores and large draft breeds with high median scores. Light horses showed mid-values [15]. Several genome-wide association studies (GWAS) have been performed for height at withers in horses [1], [2], [16], [17]. The involvement of *LCORL* (*ligand-dependent nuclear receptor compressor-like protein*) in height at withers has been primarily shown in a GWAS in Hanoverian stallions [16]. A highly significant QTL on horse chromosome (ECA) 3 in the region of *LCORL* was detected for conformation traits like head, neck, frame and development [17]. In Franches-Montagnes horses, quantitative trait loci (QTL) on ECA3 and 9 were significantly associated with withers height. Both associated SNPs were located in large intergenic regions [1]. In thoroughbred horses, the same locus on ECA3 was found as highly associated with body size. A scan of 48 horses from 16 different breeds revealed that four loci on ECA3, 6, 9 and 11 explain 83% of the variance for size. The highest associated SNP was located near the candidate gene *LCORL* and this finding was in line with the other analyses for withers height in horses [2], [16], [17]. In human, the candidate gene *LCORL* has been discussed to be involved in trunk and hip axis length [6]. GWAS in cattle for growth traits such as birth weight, body length, carcass weight and longissimus muscle area, revealed an association in the region of *NCAPG* (*non-SMC condensing I complex subunit*) and *LCORL*. Coding regions of the candidate genes were sequenced and revealed further associated markers [4]. In Angus cattle, polymorphisms in the candidate genes *adiponectin* (*ADIPOQ*) and *somatostatin* (*SST*) were tested for growth and carcass traits. The strongest statistical effect was detected for the SNP *ADIPOQ*:g.1596G>A, possibly influencing the anchoring of the transcription pre-initiation *transcription factor IID (TFIID)* complex and therefore affecting the stability of the initiation complex [18]. Analyses of *TFIID* in mice resulted in dwarf phenotypes with an about 50% reduced body weight. According to its multiple molecular functions, *TFIID* is considered as a central component of the transcription apparatus [19]. Furthermore, analyses showed a correlation between body weight, withers height and further size measurements [20].

In thoroughbred horses, a mass to height at the withers ratio was used to test the influence of the *MSTN* (*myostatin*) gene g.66493737C>T genotypes on body mass. Sprinters are proposed to be generally shorter animals with greater muscle mass [21], [22].

The objective of our study was to investigate the role of the candidate gene *LCORL* and its flanking polymorphisms with the development of body size in horses. First, we validated the association of the genomic region around *LCORL* in Hanoverian warmblood horses as well as substantiated the association of this locus with body size in a large number of horse breeds with extreme size and then we sequenced genomic and copy DNA of *LCORL* for polymorphism detection. In order to show a functional relationship of *LCORL* with body size and the associated SNP upstream of *LCORL*, we employed expression analyses for *LCORL* and its two adjacent genes for horses with differing height at withers within and across breeds.

## Results

### Within-Breed analysis

**Figure 1:**
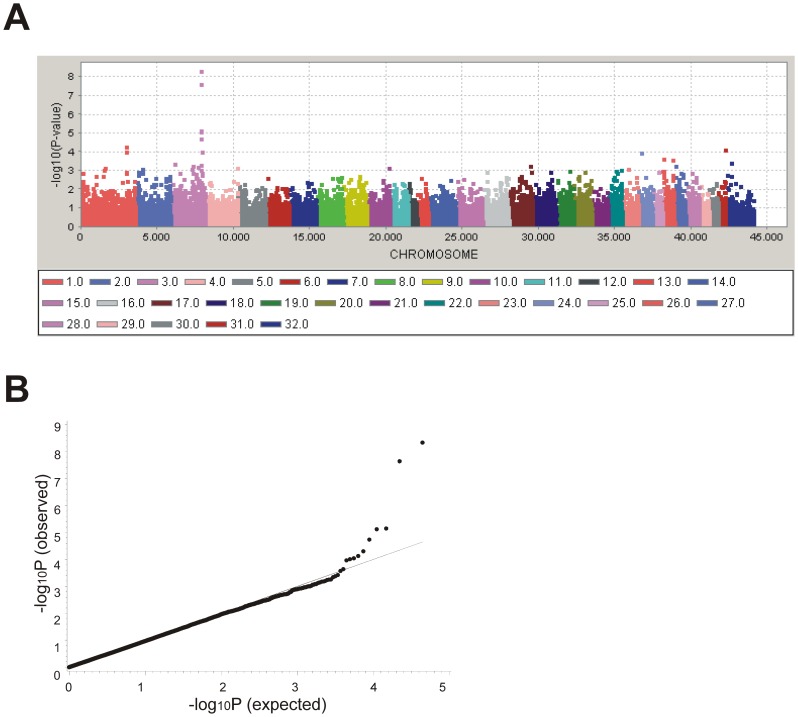
Genome-wide association analysis shows a highly significant peak on equine chromosome (ECA) 3 in Hanoverian. (A) Manhattan-plot of the −log_10_P-values from genome-wide association analysis (MLM) of body size in Hanoverian warmblood horses. The highest peak is located at 105 Mb on ECA3. (B) Q-Q plot of observed versus expected −log_10_P-values from a genome- wide association study (GWAS) in Hanoverian warmblood horses. The expected distribution (solid line) and the observed −log_10_P-values plotted against the expected −log_10_P-values (black dots) are shown. The peak value (BIEC2-808543) is located on horse chromosome 3 at 105.55 Mb.

GWAS confirmed the association of BIEC2-808543 (Broad institute nomenclature; EquCab2_105547002T>C) on ECA3 adjacent to the candidate gene *LCORL* for the Hanoverian warmblood population (Figure 1A). The observed −log_10_P-values were plotted against the expected −log_10_P-values. The quantile-quantile-plot (Q-Q) indicated that the population stratification was eliminated through the identity-by-state (IBS) kinship matrix as far as possible (Figure 1B). MLM analysis revealed the highly associated SNP BIEC2-808543 at 105.55 Mb (−log_10_P = 8.3, after accounting for multiple testing using a Bonferroni-correction −log_10_P = 3.6). The next highest associated SNP (BIEC2-808466) was located in the same region at 105.16 Mb (−log_10_P = 7.6, after accounting for multiple testing using a Bonferroni-correction −log_10_P = 2.95). The highly associated SNP BIEC2-808543 showed a minor allele frequency (MAF) of 0.45 and was distributed almost evenly among the Hanoverian warmblood horses (Table 1). In total, 35 horses were homozygous T/T and 56 horses homozygous C/C, 123 horses showed the heterozygous genotype (C/T). The distribution of the breeding values showed that BIEC2-808543 had a highly significant additive effect on the breeding value for body size, while the dominant effect approached zero.

**Table 1:** Distribution of genotypes of the associated single nucleotide polymorphism (SNP) BIEC2-808543 in 214 Hanoverian warmblood horses and the means of the breeding values with their standard errors per genotype.

Genotype	Number of animals	Frequency (%)	Standard error	Least square mean	Standard error
T/T	35	16.36	0.02	83.86	3.41
C/T	123	57.48	0.02	100.69	1.76
C/C	56	26.17	0.02	116.42	2.61

The SNP BIEC2-808543 is located at 105.55 Mb on horse chromosome (ECA) 3 with a minor allele frequency (MAF) of 0.45. The additive effect of the BIEC2-808543 polymorphisms amounted to 16.3 and the dominance effect to 0.6.

### Across-breed analysis

**Figure 2:**
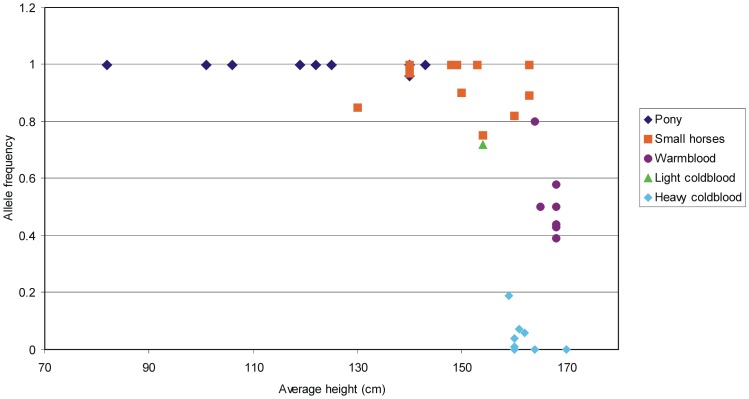
Averaged height at the withers plotted against the frequency of the allele T in ponies, small horses, light coldblood and heavy coldblood horses. The results show that the allele frequency corresponds to the average height.

After validating the highly associated SNP BIEC2-808543 in the Hanoverian warmblood population, we performed a large scan for the genotypic distribution in 1851 horses of extreme size. Genotyping of the BIEC2-808543 showed that the genotype T/T was nearly perfectly associated with all pony breeds up to the limit value of 148 cm for the height at the withers. They showed a significantly higher allele frequency for T in contrast to the larger horses (Figure 2). The small horses varying between 130 cm (Dülmener) and 160 cm in the height at withers, showed homozygous T/T and heterozygous genotypes while larger and heavier horses predominantly showed the genotype C/C (Table S1). Heterozygous horses in pony breeds showed relatively high withers height values and those stallions served breeders to pass on larger height at the wither to the offspring. The results show that this SNP proved to be a highly predictive marker of genetic potential for body size.

### Candidate region analysis

Bioinformatic sequence analysis of the genomic region of the highly associated SNP (BIEC2-808543) using Patch 1.0 and SIGNAL SCAN showed that the polymorphism was located in a putative DNA consensus sequence element, the transcription factor binding site of *TFIID*, which influences the transcription by RNA polymerase II [23], [24]. This putative TATA box element (3′-ATAAA-5′) is modified due to the mutated allele C and prediction software suggests that the putative binding site disappears because of this BIEC2-808543 mutation.

**Figure 3:**
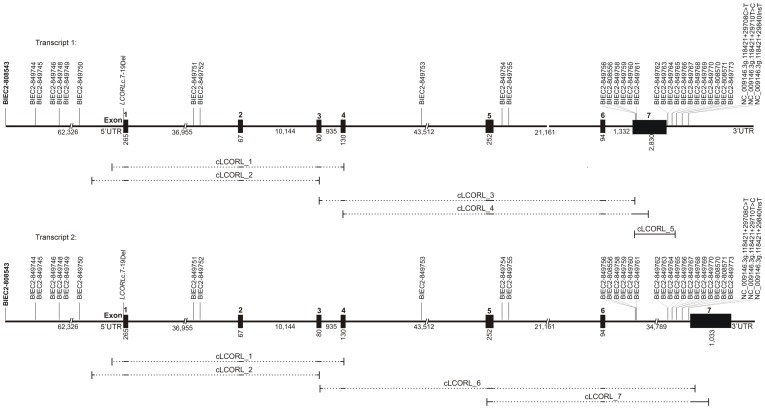
Gene model of the candidate gene *LCORL*. Sequence analyses of *LCORL* revealed two transcripts with seven exons each. The scale is in base pairs (bp). Black boxes indicate consecutively numbered exons with given sizes in base pairs (bp). Dotted and solid lines below the gene models represent the coverage of the complementary DNA (cDNA) primers. Polymorphisms are shown above and the highly associated single nucleotide polymorphism (SNP) BIEC2-808543 is printed in bold type.

*TFIID* has been supposed to play a role in influencing genes responsible for skeletal development. According to its function and its close proximity to the associated SNP, we sequenced the candidate gene *LCORL*, located on ECA3, for polymorphisms influencing body size. Comparison of the annotations of *LCORL* using NCBI (National Center for Biotechnology Information) and Ensembl resulted in two different gene models showing two transcripts with six exons with differences in the sequence of exon 6 (Ensembl) and one transcript with seven exons (NCBI). Sequence analyses of the cDNA revealed two transcripts with seven exons each (Figure 3) and showed an exon 1 sequence different to that predicted in the reference sequence. The experimental protein sequence 1 (BankIt1561108 Seq1 JX515275) showed a similarity of 97% to the human isoform 1. For the experimental protein sequence 2 (BankIt1561108 Seq2 JX515276), a similarity of 95% could be shown.

Comparing the experimental cDNA sequences of two horses with the BIEC2-808543 genotype T/T and C/C revealed a twelve base pair deletion (*BankIt1561108 Seq1 JX515275c.*7-19Del) (Table S2) in the T/T horse. Further genotyping of 71 horses of different heights and genotypes showed only eight horses with this 12-bp-deletion and did not confirm an association with body size. All experimental sequences showed various differences to the reference sequence of exon 1. These new sequences were useful to complete the annotation of the protein structure of LCORL. Sequence analyses of the genomic DNA (gDNA) revealed two SNPs (NC_009146.3g.118421+29708C>T; NC_009146.3g.118421+29710T>C) and one insertion (NC_009146.3g.118421+29840InsT) in intron 6 in comparison with the reference sequence (Table S2) but these polymorphisms were not associated with body size.

### Expression study

**Figure 4:**
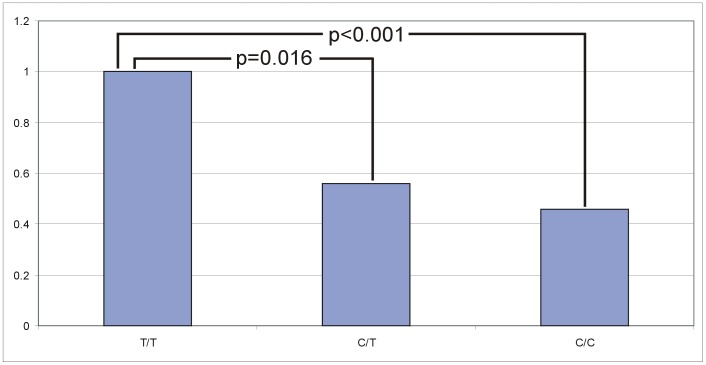
Relative expression levels of *LCORL* in relation to the BIEC2-808543 genotype across five different breeds. In comparison with the T/T genotypes, the expression of horses with the C/T genotype (P = 0.016) is decreased by 44% and for the genotype C/C (P<0.001) by 54%. The expression differences were accounted for using the ΔΔCT method.

**Figure 5:**
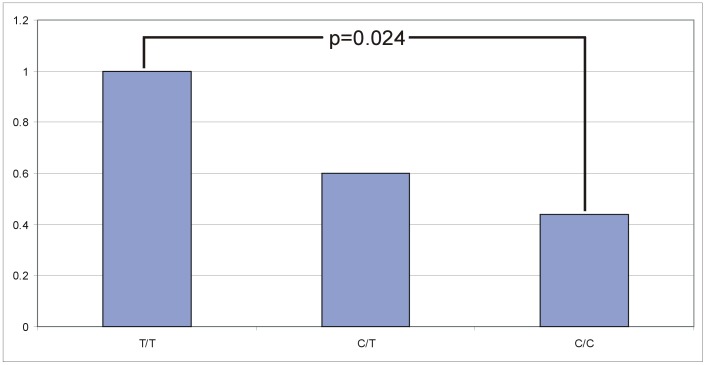
Relative expression levels of *LCORL* in relation to the BIEC2-808543 genotype in 13 Hanoverian horses. Using the T/T genotype as standard, the expression of horses with the C/T genotype is decreased by 40% and for the genotype C/C (P = 0.024) by 56%. The expression differences were accounted for using the ΔΔCT method.

The quantitative real-time PCR (RT-qPCR) analysis for *LCORL* revealed a significant decrease of relative *LCORL* expression in medium sized horses (Hanoverian 166–168 cm and Dülmener >133 cm) with the genotype C/T and an even more decreased relative expression in larger sized horses with the SNP genotype C/C (Hanoverian >168 cm and Rhenish German Draught). Across all horses analysed, a significant decrease of 40% could be shown in heterozygous (C/T) (P = 0.016) and 56% in homozygous C/C horses (P<0.001) in comparison to T/T horses (Figure 4). In the Hanoverian warmblood population, the relative expression of the heterozygous C/T-horses was decreased by 44% and in homozygous C/C-horses by 54% (P = 0.024) when T/T-horses were used as reference (Figure 5). General linear model (GLM) analysis revealed a significant effect for the T>C genotype but neither a significant influence of the sex, breed or breed by genotype on the expression results. Furthermore, the age and time of sampling had also no effect on the different expression levels. Relative expression analyses of the adjacent genes *NCAPG* and *DCAF16 (damage-specific DNA-binding protein and cullin-4 associated factor 16)* revealed no significant effects associated with body size or the T>C genotypes using GLM analyses (Figures S1, S2). Semi-quantitative expression analyses of testis, hair, brain, kidney, muscle and liver tissues revealed that *LCORL*, *NCAPG* and *DCAF16* are almost equally expressed in these tissues and hair roots (Figure S3). EST profiles confirmed that these genes are almost ubiquitously expressed (http://www.ncbi.nlm.nih.gov/UniGene).

## Discussion

In this study, we could show that the T>C mutation of the SNP BIEC2-808543 is located within a potential transcription factor binding site and this mutation is significantly associated with relative expression levels of *LCORL* and simultaneously with body size within and across horse breeds. GWAS within Hanoverian warmblood horses and across a large number of horses from 42 different breeds confirmed this SNP to be a highly predictive marker for body size.

The body size associated SNP is located within a binding site for the general transcription factor *TFIID* working as a TATA box-binding factor. While the homozygous genotype T/T of BIEC2-808543 possibly enables the anchoring of *TFIID*, the SNP allele C could presumably modify the binding site function and therefore influence the *TFIID* recognition of core promoter elements, the first step of the initiation of mRNA transcription [24]. This initiation is a key stage in the regulation of gene expression and plays an important role in the regulation of the AP-1, *activator protein-1 transcription factor complex*, in bone cell development [25], [26]. Skeletal bones are developed by the three cell types, chrondrocytes, osteoblasts and osteoclasts. The differentiation and function of theses cells is regulated by several factors influencing specific gene expression. Analyses in human bone have shown that members of the *AP-1* family strongly affect these cells [27], [28]. In vivo studies in mice with *TFIID* inactivated component resulted in a reduced size of different organs including an attenuated growth, resulting in dwarfism with an about 50% reduced body weight, and showed that a lacking function of the *TFIID* mechanism is able to have a strong influence on body size [19].

Modifications in transcriptional regulation are proposed to influence several biological processes determining body size. Possible targets discussed in adult height analysis were genes controlling intracellular signalling, cell division, DNA replication and skeletal development [3], [6], [29], [30]. Therefore, the C allele of BIEC2-808543 is presumably the reason for the reduced expression of *LCORL* in larger sized horses. *LCORL*, also known as the *Mblk1-related protein*, shows characteristic motifs of transcription factors and analyses with mouse tissues indicate that it is able to activate transcription. Transcription factors are key proteins in various biologic processes. To achieve their different roles they have to change or specialize their functions or target genes [31]. We assume that this specific function of *LCORL* could possibly be involved in such a complex trait.

In human genome-wide scans for adult stature evidences *LCORL* to be associated with trunk length and hip axis length [6]. According to the conserved synteny of 53% of the equine chromosomes to a single human chromosome [32] horses' size could possibly be influenced by similar genomic regions. In cattle, a GWAS revealed two SNPs in *LCORL* highly associated with feed intake and body weight gain phenotypes. It was supposed that SNPs affecting the transcription or translation of *LCORL* may result in an increased or decreased regulation of genes involved in growth [4].

We assume that similar effects might also explain the variation in body sizes in horses. GWAS for body size in horses in previous studies revealed several QTL for different breeds [1], [2], [16], [17]. Depending on breed and population different candidate genes were discussed. Nevertheless, all studies had the QTL on ECA3 near *LCORL* in common [1], [2], [16], [17]. The present analysis of the Hanoverian warmblood horses and extreme size horse breeds confirmed the associated SNP adjacent to *LCORL* and emphasized the assumption that *LCORL* is strongly involved in the development of body size within and across breeds. This finding was the basis for the gene expression analysis.

The expression analysis was performed using hair root samples due to the ubiquitous expression profile and the non invasive sampling of these tissues. Body size is assumed to be a result of different gene interactions in human that are not yet investigated [5], [6]. Nevertheless, the correlation between growth of body and hair could be shown in various studies. The Rothmund-Thomson syndrome in human for example is characterized by severe dwarfism combined with an abnormal hair growth [33]. Studies in mice revealed growth retardation in hair length and a retarded rate of body growth caused by the supply of high concentrations of the epidermal growth factor (EGF) [34].

Our results of the expression analyses showed that the relative expression levels of *LCORL* decreased considerably in larger sized and heavy horses with the C/C genotype. Horses heterozygous for the SNP BIEC2-808543 showed relative expression levels in-between the two homozygous genotypes. They represent the medium sized horses and producers of larger sized ponies. The mechanism how BIEC2-808543 effects *LCORL* is not yet known but the mutated transcription factor binding site might cause significant changes of the relative expression levels.

The distribution of body sizes and expression levels indicated that *LCORL* might act as a main regulator for body size. Nevertheless, as studies in human height suggest, body size is a complex trait and further regulatory elements must be involved to create such huge differences in skeletal morphologic types [15]. The candidate genes *NCAPG* and *DCAF16* could be eliminated as candidates, whereas *HMGA2* (*high mobility group AT-hook 2*, ECA6), *ZFAT* (*zinc finger and AT hook domain containing*, ECA9), *LASP1* (*LIM and SH3 protein 1*, ECA11) could also possibly involved.

The results demonstrate that we have identified a functional polymorphism differentiating ponies from medium and large and heavy horse breeds and accounting for body size variation within horse breeds. This T>C polymorphism is located within the binding site of the transcription factor *TFIID* and was significantly associated with body size and relative expression levels of *LCORL*. This is the first functional study for a body size regulating polymorphism in horses and further steps are necessary to unravel the mechanisms for understanding the complex genetic regulation of body size in horses.

## Materials and Methods

### Ethics statement

All animal work has been conducted according to the national and international guidelines for animal welfare. The EDTA-blood and hair root sampling was approved by the Lower Saxony state veterinary office, Niedersächsisches Landesamt für Verbraucherschutz und Lebensmittelsicherheit, Oldenburg, Germany (registration numbers 02A-138 and 07A-482).

### Animals

Blood samples were collected from 214 Hanoverian warmblood horses including 150 stallions of the National State Stud of Lower Saxony and 64 broodmares. For these horses, breeding values, conformation and pedigree data were made available by the Hanoverian Studbook Society (HSS) through the national unified animal ownership database (Vereinigte Informationssysteme Tierhaltung w.V., VIT, Verden/Aller, Germany). We used the latest breeding values (BVs) for height at withers (WH) (edited in November 2011) provided by HSS. BVs for WH were estimated based on results of studbook inspection (SBI) since 1979 including 85,598 Hanoverian warmblood horses. BVs are estimated yearly through the VIT for WH employing a BLUP (best linear unbiased prediction) animal model [35].
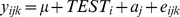
with *y_ijk_* = height at withers, *μ* = model constant, *TEST_i_* = fixed effect of the individual test representing the interaction between the place, year and season of performance evaluation, *a_j_* = random additive genetic effect of the individual horse and *e_ijk_* = random residual.

All BVs were standardized to a mean value of 100 points and a standard deviation of 20 points using the horses of the birth cohorts from 2000 and 2002 as reference.

For the horses studied here, the mean BV for WH was 105±25 (range 24–164). The mean reliabilities of the BVs were at 0.9. The distribution of the BVs for WH was analysed using the UNIVARIATE procedure of SAS (Statistical Analysis System, version 9.3, SAS Institute, Cary, NC, USA, 2011). The additive genetic effect was estimated as half of the difference of the least square means among the two homozygous genotypes. The dominance effect was calculated as the deviation of the least square mean of the heterozygotes from the average of the two homozygous genotypes.

For each horse the proportions of genes of Hanoverian (HAN), Thoroughbred (TB), Trakehner (TRAK) and Holsteiner (HOL) were calculated using all available pedigree information. Details are described elsewhere [36]. Mean (median) proportions of genes in the stallions were 0.54 (0.63) for HAN, 0.28 (0.19) for TB, 0.05 (0.03) for TRAK, and 0.06 (0) for HOL.

Across-breed analysis was performed in 1851 horses of fourty-two different breeds including the pony breeds American Miniature Horse, Dartmoor, Exmoor, German Classic Pony, German Riding Pony, Haflinger, Icelandic, Lewitzer Pony, Miniature Shetland pony, Norwegian Fjord Horse, Shetland pony, Terceira Pony, Welsh Section A and the horse breeds with average withers height values below 148 cm (58.27 inches) (Dülmener, Arabian, Przewalski, Sorraia) or a range of variation around 148 cm (58.27 inches) (Anglo-Arabian, American Paint Horse, Appaloosa, Black Forest Horse, Lusitano, Peruvian Paso, Quarter Horse, Tinker, Thoroughbred), lighter coldblood horses (Black Forest Horse), heavy draft horse breeds (Altmark Coldblood, Mecklenburg Coldblood, Noriker horse, Rhenish German Draught, Saxon Thuringian Coldblood, Schleswig Draught, Shire Horse, South German Coldblood) and warmblood horse breeds (Hanoverian, Oldenburg, Westphalian, Rhinelander horse, Trakehner, Zweibrücker, Holsteiner, Selle Francais). Size ranges were detected for every breed and results were averaged.

### Genotyping

Genomic DNA was isolated using 500 µl EDTA blood by standard ethanol fraction. Precipitation was achieved by 6 M NaCl, 70% ethanol, and 100% ethanol (Carl Roth) in consecutive steps according to standard protocols. Genotyping was performed with the Illumina equine SNP50 BeadChip (Illumina, San Diego, CA, USA) including 54,602 SNPs using standard procedures as recommended by the manufacturer. Data were analyzed and file clusters were generated using the genotyping module version 3.2.32 of the BeadStudio program (Illumina).

Across breed analysis required an additional genotyping of BIEC2-808543 by the use of restriction fragment length polymorphism (RFLP). We used the restriction enzyme BsrI according to NEBcutter V2.0 recommendations (http://tools.neb.com/NEBcutter2/) and primers were designed using Primer3 (http://frodo.wi.mit.edu/primer3/) (Table S3). The reaction was assembled in 30 µl total volume containing 2 µl DNA, 17.6 µl H_2_O, 6.4 µl enhancer solution P (Peqlab Biotechnologie), 3.2 µl incubation mix with MgCl2, 0.5 µl dNTP mix, 0.3 µl 1000 U Taq Polymerase (Taq Core Kit 10 (1000 U), MP Biomedicals, LLC, Germany), 1 µl forward and 1 µl reverse primers. The reaction was performed on a PTC 200™ thermocycler (MJ Research, Inc., Waltham, USA) setting 30 seconds denaturation at 94°C, 34 cycles of 94°C for 30 seconds, 60°C annealing temperature, 72°C for 40 seconds and finally 4°C for 10 minutes. The incubation of PCR (polymerase chain reaction) amplificates (10 µl) was performed with 16.75 µl H2O and 3 µl NEB buffer 3 (New England Biolabs, Ipswich, USA) at 65°C for 12 hours. Products were separated by gel electrophoresis using 3% agarose gels (peqGold MoSieve Agarose MS 500, Peqlab Biotechnologie). Genotypes were determined by visual examination under UV illumination (BioDocAnalyze, Biometra, Göttingen, Germany).

The polymorphism *BankIt1561108 Seq1 JX515275c.*7-19del was genotyped in 71 horses including one Arabian, one Dartmoor, twenty-four German Classic Ponies, eight Haflinger, ten Hanoverian, one Icelandic, three Miniature Shetland ponies, one Noriker horse, one Norwegian Fjord Horse, one Oldenburg horse, eight Rhenish German Draught, seven Shetland ponies and five Welsh Section A. Fluorescence labelled primer 5′-AGGGCTCCGGCACTGAGCAG-3′ and unmodified primer 5′-CAGAGGGAAGGTAGTGACACG-3′ were used for PCR-amplification with an annealing temperature of 60°C according to standard protocols. PCR products were size-fractioned by gel electrophoresis on 6% polyacrylamide denaturing gels (RotiphoreseGel 40, Carl Roth) using an automated capillary sequencer (LI-COR 4200/S-2, LI-COR 4300, LI-COR Biotechnology, Bad Homburg, Germany).

### Candidate region analysis

Bioinformatic analysis of the region of BIEC2-808543 was performed using Patch 1.0 (http://www.gene-regulation.com/cgi-bin/pub/programs/pmatch/bin/p-match.cgi) on the public database TRANSFAC (version 7.0, Public 2005) which contains data on transcription factors, their experimentally-proven binding sites, and regulated genes. Further verification was done by SIGNAL SCAN (http://www.gene-regulation.com/cgi-bin/pub/programs/sigscan/sigscan.cgi). The model of the candidate gene *LCORL* was build using Spidey (http://www.ncbi.nlm.nih.gov/spidey/index.html), a tool to align expressed sequences to their parent genomic sequences [37]. For BLAST searches, the resources of the National Center for Biotechnology Information (NCBI) were used (http://www.ncbi.nlm.nih.gov/blast/Blast.cgi) [38]. The open reading frame (ORF) Finder (http://www.ncbi.nlm.nih.gov/projects/gorf/) and Windows 32 EditSeq 4.03, graphical analysis tools which find all open reading frames of a selectable minimum size in a user's sequence, were also used for analysis.

Equine complementary DNA (cDNA) of the candidate gene *LCORL* (NCBI Gene ID: 100068577; Ensembl ENSECAG00000000648) was sequenced in three German warmblood horses and one Arabian thoroughbred using hair root, kidney and testicular tissues. RNeasy Lipid Tissue Mini Kit (Qiagen, Hilden, Germany) was used for purification of about 50 µg total RNA from stabilized tissues according to manufacturer's protocol. It was transcribed into cDNA by Maxima First Strand cDNA Synthesis Kit for RT-qPCR (Fermentas Life Sciences, St. Leon-Rot, Germany). Quality control was performed with primers (5′CAAAAACAACAGACAGCCTTATGC-3′ and 5′-GCTCTGCCAGTACCCCAAGA-3′) of the *RPL4, ribosomal protein L4*, gene (ECA1) spanning two exons and a short intron in between. The product size differed clearly in cDNA (80 bp) and gDNA (268 bp) products. Primers were designed using Primer3 (http://frodo.wi.mit.edu/primer3/) (Table S4) and PCR was performed in 20 µl total volume. We used 2 µl cDNA, 12.1 µl H_2_O, 4.2 µl enhancer solution P (Peqlab Biotechnologie), 2.1 µl incubation mix with MgCl2, 0.3 µl dNTP mix, 0.3 µl 1000 U Taq Polymerase (Taq Core Kit 10 (1000 U), MP Biomedicals, LLC, Germany) and 0.5 µl forward and 0.5 µl reverse primers. The reaction was performed on a PTC 200™ thermocycler (MJ Research): 4 minutes denaturation at 94°C, followed by 34 cycles of 94°C and primer adapted annealing temperature for 30 seconds, 72°C for 40 seconds and finally 4°C for 10 minutes. Furthermore, except for the first exon, the exons and exon/intron boundaries of *LCORL* were sequenced on gDNA in ten horses to identify polymorphisms. PCR conditions were according to the cDNA analysis. Three horses showed the BIEC2-808543 genotype C/C, one horse was homozygous for T and seven horses had a heterozygous genotype (C/T). In exon 1 no appropriate PCR product could be amplified while nine of ten sequences of exon two didn't have sufficient quality for the evaluation. Sequencing was performed by the automated sequencer Genetic Analyzer 3500 (Applied Biosystems by Life Technologies).

### Expression analysis

For the analysis of the *LCORL*, *NCAPG* and *DCAF16* expression, RNA was isolated from hair root samples of eleven Arabian and three Welsh Section A with the genotype T/T, eight Rhenish German Draught with the genotype C/C, twelve Dülmener showing the genotypes T/T and C/T and 13 Hanoverian of all three genotypes by RNeasy Lipid Tissue Mini Kit (Qiagen) according to manufacturer's protocol (Table S5). Furthermore we isolated testis, brain, kidney, muscle and liver tissues as controls for semi-quantitative expression analysis. It was transcribed into cDNA by Maxima First Strand cDNA Synthesis Kit for RT-qPCR (Fermentas Life Sciences). Glyceraldehyde-3-phosphate dehydrogenase (*GAPDH*) was used as a housekeeping gene. The reactions were run on an optical 96-well reaction plate (Applied Biosystems) in a final volume of 11.5 µl containing 1.5 µl cDNA, 1.0 µl reverse and 1.0 µl forward primes of the candidate gene and the *GAPDH* (Table S6), 0.25 µl VIC-labeled TaqMan probe for the candidate gene and FAM-labeled TaqMan probe for *GAPDH* (Applied Biosystems), 1.68 µl nuclease free water and 6 µl Maxima Probe qPCR master mix 2× supplemented by 0.07 µl ROX Solution (Fermentas Life Sciences). The quantitative real-time (qRT)-PCR was performed using an ABI7300 sequence detection system (Applied Biosystems) under the following conditions: 10 min at 95°C followed by 40 cycles at 95°C for 15 sec and 60°C for 1 min. All samples were analysed in duplicates. The identified quantities of gene expression were normalised by the *GAPDH* expression level (ΔCT). For calibration the average ΔCT of all horses with the genotype T/T was used and the relative expression level was calculated by the ΔΔCT method [39]. The average ΔCT of medium sized horses (C/T) and of larger horses (C/C) was subtracted from the standard ΔCT (T/T) and the potency was calculated from these values. The deviations from the standard ( = 1) were given as a percentage. The same procedure was applied for the Hanoverians.

We performed a GLM analysis using SAS/Genetics, version 9.3 (Statistical Analysis System, 2012) for testing the effects of genotype, sex, breed, breed by genotype as well as age and time of sampling. Semi-quantitative expression analysis of different tissues was performed by PCR, using the primer pairs of qPCR analysis. The reaction was assembled in 20 µ, including 2 µl cDNA, 12.1 µl H2O, 4.2 µl enhancer solution P (Peqlab Biotechnologie), 2.1 µl incubation mix with MgCl2, 0.3 µl dNTP mix, 0.3 µl 1000 U Taq Polymerase (Taq Core Kit 10 (1000 U), MP Biomedicals) and 0.5 µl forward and 0.5 µl reverse primers. The reaction was performed on a PTC 200™ thermocycler (MJ Research): 4 minutes denaturation at 94°C, followed by 30 cycles of 94°C and 60°C for 30 seconds, 72°C for 40 seconds and finally 4°C for 10 minutes.

### Data analysis

Analysis was performed in 44,496 SNPs with a minor allele frequency (MAF) >0.05 and a call rate of >90%. The data quality control was done using PLINK, version 1.07 (http://pngu.mgh.harvard.edu/purcell/plink/) [40] and SAS/Genetics, version 9.3 (2012). For the GWAS, a mixed linear model (MLM) was employed in order to control data for stratification. A marker based identity-by-state (IBS) kinship matrix among all horses (K-matrix) was employed for parameterization of a random polygenic effect. Fixed effects were the sex of the animal and gene proportions of Hanoverian, Thoroughbred, Holsteiner and Trakehner using TASSEL (Trait Analysis by Association, Evolution and Linkage) version 3.0, a software package for association mapping of complex traits in diverse samples [41]. Quantile-quantile (Q-Q) plots for observed versus expected −log_10_P-values were constructed to control for population stratification. Significance thresholds were determined using a Bonferroni correction and the MULTITEST procedure of SAS.

## Supporting Information

Figure S1 Relative expression level of *NCAPG* in relation to the BIEC2-808543 genotype across five different breeds (A) and within-breed in 13 Hanoverian horses (B). No significant differences between the expression levels of horses of different sizes and genotypes could be seen.
(DOC)

Figure S2 Relative expression level of *DCAF16* in relation to the BIEC2-808543 genotype across five different breeds (A) and within-breed in 13 Hanoverian horses (B). No significant differences between the expression levels of horses of different sizes and genotypes could be seen.
(DOC)

Figure S3 Semi-quantitative PCR analysis of investigated genes in different tissues. The expression of testis, hair roots, brain, kidney, muscle and liver is shown for *LCORL* (A), *NCAPG* (B) and *DCAF16* (C).
(DOC)

Table S1 Number of animals genotyped for the SNP BIEC-808543 on horse chromosome 3, average height at withers and genotypic distribution per breed.
(DOC)

Table S2 Polymorphisms and their position, type, base change and source identified in the sequence analysis of *LCORL*. No associations for different body sizes could be detected.
(DOC)

Table S3 Single nucleotide polymorphism on equine chromosome (ECA) 3, its primer sequence and product size used for genotyping by restriction fragment length polymorphism (RFLP).
(DOC)

Table S4 **Primer sequences and their position, product size and annealing temperature (AT) for sequencing the equine genomic and cDNA of** ***LCORL*** **.**
(DOC)

Table S5 **Samples used for expression analysis of** ***LCORL*** **,** ***NCAPG*** **and** ***DCAF16*** **.** The breed, height at the withers, genotype, sex, age at the time of sampling and the time of sampling are shown.
(DOC)

Table S6 **Primer sequences, their product sizes, annealing temperatures (AT) and TaqMan probes used for real-time quantitative PCR (RT-qPCR) for** ***LCORL*** **,** ***NCAPG*** **and** ***DCAF16*** **using** ***GAPDH*** **as reference gene.**
(DOC)
