# Supplementary material for: Expression Levels of LCORL Are Associated with Body Size in Horses
Source: PLoS One. 2013 Feb 13;8(2):e56497. doi: 10.1371/journal.pone.0056497 (PMC3572084; doi:10.1371/journal.pone.0056497)
Supplement: Figure S2 — Relative expression level of DCAF16 in relation to the BIEC2-808543 genotype across five different breeds (A) and within-breed in 13 Hanoverian horses (B). No significant differences between the expression levels of horses of different sizes and genotypes could be seen. (DOC) [file pone.0056497.s002.doc]

**
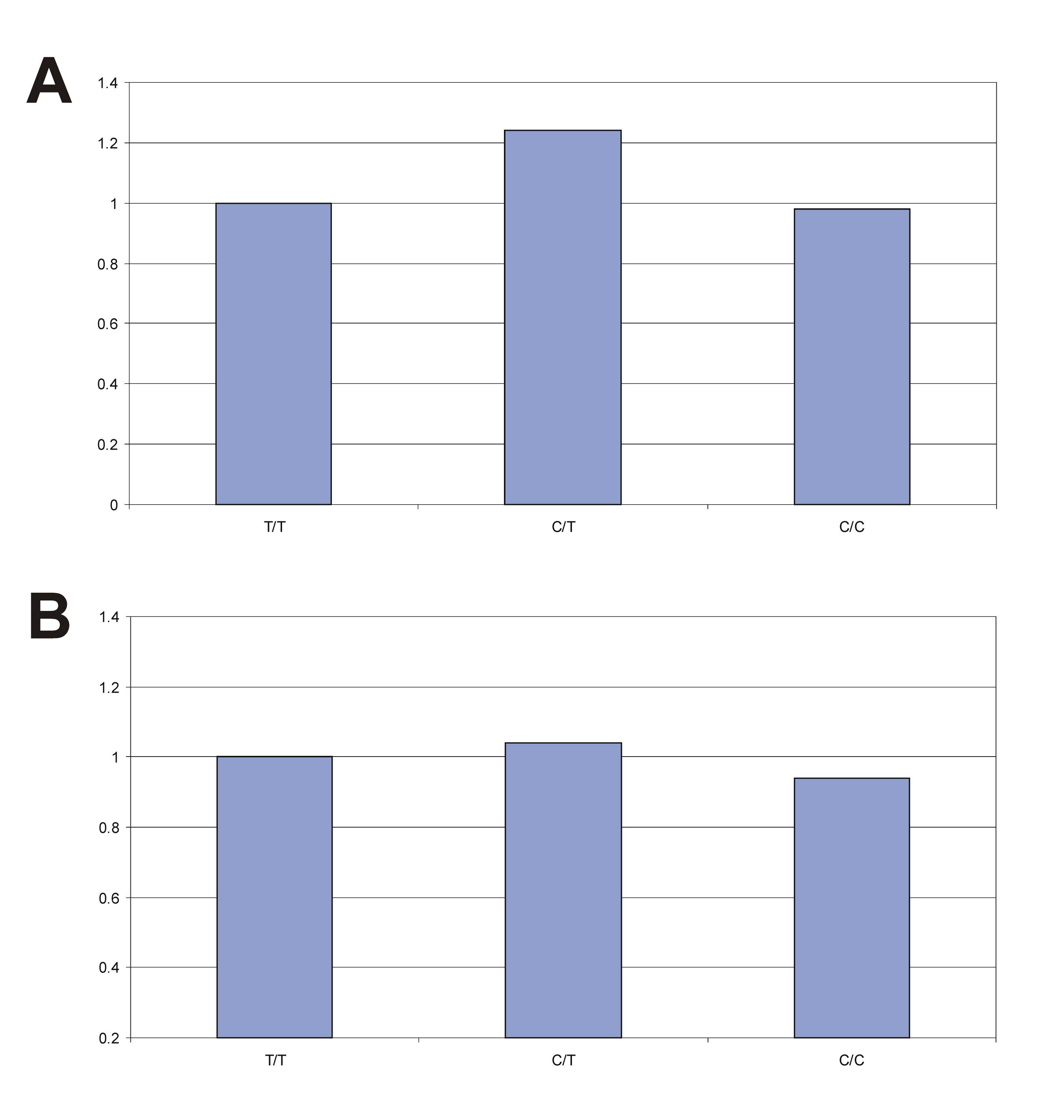
**

**Figure S2. Relative expression level of *DCAF16* in relation to the BIEC2-808543 genotype across five different breeds (A) and within-breed in 13 Hanoverian horses (B).** No significant differences between the expression levels of horses of different sizes and genotypes can be seen.
