# Supplementary material for: Expression Levels of LCORL Are Associated with Body Size in Horses
Source: PLoS One. 2013 Feb 13;8(2):e56497. doi: 10.1371/journal.pone.0056497 (PMC3572084; doi:10.1371/journal.pone.0056497)
Supplement: Figure S3 — Semi-quantitative PCR analysis of investigated genes in different tissues. The expression of testis, hair roots, brain, kidney, muscle and liver is shown for LCORL (A), NCAPG (B) and DCAF16 (C). (DOC) [file pone.0056497.s003.doc]

**
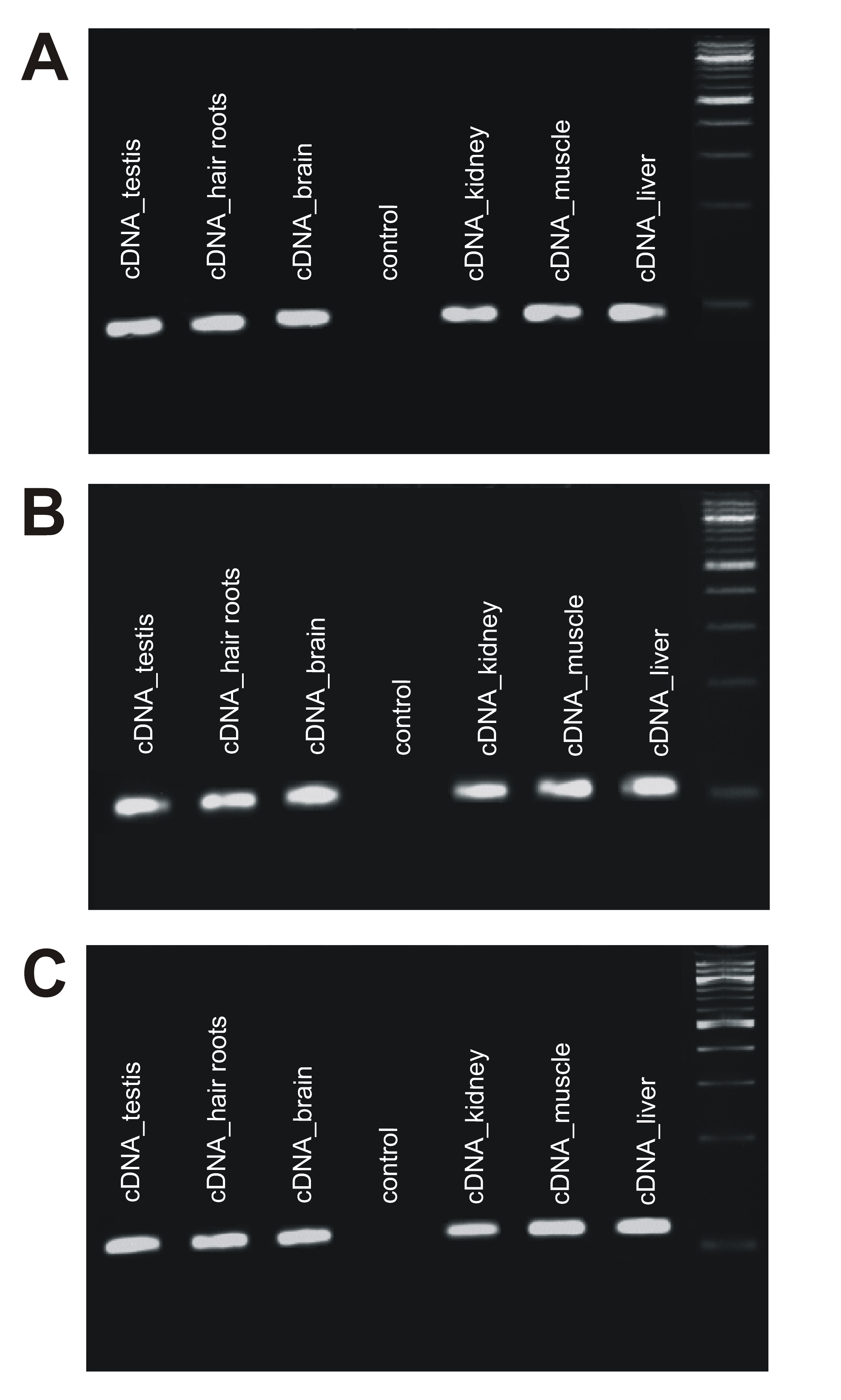
**

**Figure S3. Semi-quantitative PCR analysis of investigated genes in different tissues.** The expression of testis, hair roots, brain, kidney, muscle and liver is shown for *LCORL* (A), *NCAPG* (B) and *DCAF16* (C).
