# Supplementary material for: Expression Levels of LCORL Are Associated with Body Size in Horses
Source: PLoS One. 2013 Feb 13;8(2):e56497. doi: 10.1371/journal.pone.0056497 (PMC3572084; doi:10.1371/journal.pone.0056497)
Supplement: Table S1 — Number of animals genotyped for the SNP BIEC-808543 on horse chromosome 3, average height at withers and genotypic distribution per breed. (DOC) [file pone.0056497.s004.doc]

**Table S1.** **Number of animals genotyped for the SNP BIEC-808543 on horse chromosome 3, average height at withers and genotypic distribution per breed.**

| Breed | Height at withers (cm) | Frequency (n) | Frequency (%) | Genotypic distribution  T/T C/T C/C | | |
| --- | --- | --- | --- | --- | --- | --- |
| **Pony** |  |  |  |  |  |  |
| American Miniature Horse | 82 | 1 | 0.05 | 1.00 | 0.00 | 0.00 |
| Miniature Shetland Pony | 82 | 3 | 0.16 | 1.00 | 0.00 | 0.00 |
| Shetland pony | 101 | 9 | 0.48 | 1.00 | 0.00 | 0.00 |
| German Classic Pony | 106 | 25 | 1.34 | 1.00 | 0.00 | 0.00 |
| Welsh Section A | 119 | 5 | 0.27 | 1.00 | 0.00 | 0.00 |
| Dartmoor | 122 | 1 | 0.05 | 1.00 | 0.00 | 0.00 |
| Exmoor | 125 | 20 | 1.07 | 1.00 | 0.00 | 0.00 |
| Terceira | 125 | 13 | 0.70 | 1.00 | 0.00 | 0.00 |
| Icelandic | 140 | 53 | 2.84 | 1.00 | 0.00 | 0.00 |
| Lewitzer Pony | 140 | 56 | 3.00 | 0.93 | 0.07 | 0.00 |
| German Riding Pony | 143 | 4 | 0.21 | 1.00 | 0.00 | 0.00 |
| Haflinger | 143 | 14 | 0.75 | 1.00 | 0.00 | 0.00 |
| Norwegian Fjord Horse | 143 | 24 | 1.29 | 1.00 | 0.00 | 0.00 |
| **Donkey** |  |  |  |  |  |  |
| Donkey | 125 | 15 | 0.80 | 1.00 | 0.00 | 0.00 |

**Table S1. continued**

| Breed | Height at the withers | Frequency (n) | Frequency (%) | Genotypic distribution  T/T C/T C/C | | |
| --- | --- | --- | --- | --- | --- | --- |
| **Small horses** |  |  |  |  | | |
| Dülmener | 130 | 80 | 4.29 | 0.70 | 0.30 | 0.00 |
| Sorraia | 140 | 33 | 1.77 | 0.94 | 0.06 | 0.00 |
| Przewalski | 140 | 17 | 0.91 | 1.00 | 0.00 | 0.00 |
| Arabian | 148 | 223 | 11.95 | 1.00 | 0.00 | 0.00 |
| Tinker | 148 | 1 | 0.05 | 1.00 | 0.00 | 0.00 |
| Peruvian Paso | 149 | 4 | 0.21 | 1.00 | 0.00 | 0.00 |
| American Paint Horse | 150 | 5 | 0.27 | 0.80 | 0.20 | 0.00 |
| Quarter Horse | 153 | 9 | 0.48 | 1.00 | 0.00 | 0.00 |
| Appaloosa | 154 | 2 | 0.11 | 0.50 | 0.50 | 0.00 |
| Lusitano | 160 | 96 | 5.14 | 0.67 | 0.29 | 0.04 |
| Anglo-Arabian | 163 | 1 | 0.05 | 1.00 | 0.00 | 0.00 |
| Thoroughbred | 163 | 14 | 0.75 | 0.79 | 0.21 | 0.00 |
| **Light coldblood** |  |  |  |  |  |  |
| Black Forest Horse | 154 | 136 | 7.29 | 0.49 | 0.46 | 0.05 |

**Table S1. continued**

| Breed | Height at the withers | Frequency (n) | Frequency (%) | Genotypic distribution  T/T C/T C/C | | |
| --- | --- | --- | --- | --- | --- | --- |
| **Warmblood** |  |  |  |  |  |  |
| Trakehner | 164 | 5 | 0.27 | 0.60 | 0.40 | 0.00 |
| Zweibrücker | 165 | 1 | 0.05 | 0.00 | 1.00 | 0.00 |
| Hanoverian | 168 | 590 | 31.62 | 0.16 | 0.56 | 0.28 |
| Oldenburg horse | 168 | 21 | 1.12 | 0.14 | 0.57 | 0.29 |
| Holsteiner horse | 168 | 11 | 0.59 | 0.18 | 0.64 | 0.18 |
| Westphalian | 168 | 45 | 2.41 | 0.09 | 0.60 | 0.31 |
| Rhinelander horse | 168 | 12 | 0.64 | 0.33 | 0.50 | 0.17 |
| Selle Français | 168 | 1 | 0.05 | 0.00 | 1.00 | 0.00 |
| **Heavy coldblood** |  |  |  |  |  |  |
| Schleswig Draught | 159 | 45 | 2.41 | 0.04 | 0.29 | 0.67 |
| Mecklenburg Coldblood | 160 | 22 | 1.18 | 0.00 | 0.09 | 0.91 |
| Noriker horse | 160 | 89 | 4.77 | 0.00 | 0.11 | 0.99 |
| South German Coldblood | 160 | 45 | 4.76 | 0.00 | 0.00 | 1.00 |
| Saxon Thuringian Coldblood | 161 | 22 | 1.18 | 0.00 | 0.14 | 0.86 |

**Table S1. continued**

| Breed | Height at the withers | Frequency (n) | Frequency (%) | Genotypic distribution  T/T C/T C/C | | |
| --- | --- | --- | --- | --- | --- | --- |
| Altmark Coldblood | 162 | 32 | 1.71 | 0.00 | 0.12 | 0.87 |
| Rhenish German Draught | 164 | 61 | 3.27 | 0.00 | 0.00 | 1.00 |
| Shire Horse | 170 | 1 | 0.05 | 0.00 | 0.00 | 1.00 |
