# Supplementary material for: Expression Levels of LCORL Are Associated with Body Size in Horses
Source: PLoS One. 2013 Feb 13;8(2):e56497. doi: 10.1371/journal.pone.0056497 (PMC3572084; doi:10.1371/journal.pone.0056497)
Supplement: Table S2 — Polymorphisms and their position, type, base change and source identified in the sequence analysis of LCORL. No associations for different body sizes could be detected. (DOC) [file pone.0056497.s005.doc]

**Table S2. Polymorphisms and their position, type, base change and source identified in the sequence analysis of *LCORL*.** No associations for different body sizes could be detected.

| ECA | Gene | Polymorphism name | Type | Base change | Source (transcript 1/ 2) |
| --- | --- | --- | --- | --- | --- |
| 3 | *LCORL* | NC_009146.3g.118421+29708C>T | SNP | C>T | 3’UTR/ intron |
| 3 | *LCORL* | NC_009146.3g.118421+29710T>C | SNP | T>C | 3’UTR/ intron |
| 3 | *LCORL* | NC_009146.3g.118421+29840InsT | Insertion | T | 3’UTR/ intron |
| 3 | *LCORL* | *BankIt1561108 Seq1 JX515275*c.7-19Del | Deletion | GCTGCCGCCGCC | Exon 1 |
