# Supplementary material for: Expression Levels of LCORL Are Associated with Body Size in Horses
Source: PLoS One. 2013 Feb 13;8(2):e56497. doi: 10.1371/journal.pone.0056497 (PMC3572084; doi:10.1371/journal.pone.0056497)
Supplement: Table S3 — Single nucleotide polymorphism on equine chromosome (ECA) 3, its primer sequence and product size used for genotyping by restriction fragment length polymorphism (RFLP). (DOC) [file pone.0056497.s006.doc]

**Table S3.** **Single nucleotide polymorphism on equine chromosome (ECA) 3, its primer sequence and product size used for genotyping by restriction fragment length polymorphism (RFLP).**

| Polymorphism (Broad institute nomenclature) | Forward primer (5’-3’) | Reverse primer (5’-3’) | Product size (bp) | Restriction enzyme | Fragment sizes  Allel A Allel B | |
| --- | --- | --- | --- | --- | --- | --- |
| BIEC2-808543 | GCCATCTATTTGCATGTTCTTG | GGCAAGTTCATAGGCTGGTTC | 347 | BsrI | 235/ 57/ 55 | 292/ 55 |
