# Supplementary material for: Expression Levels of LCORL Are Associated with Body Size in Horses
Source: PLoS One. 2013 Feb 13;8(2):e56497. doi: 10.1371/journal.pone.0056497 (PMC3572084; doi:10.1371/journal.pone.0056497)
Supplement: Table S4 — Primer sequences and their position, product size and annealing temperature (AT) for sequencing the equine genomic and cDNA of LCORL . (DOC) [file pone.0056497.s007.doc]

**Table S4.** **Primer sequences and their position, product size and annealing temperature (AT) for sequencing the equine genomic and cDNA of *LCORL*.**

| Gene | Gene region | Forward primer (5’-3’) | Reverse primer (5’-3’) | Product size (bp) | AT^a^ (°C) | Name |
| --- | --- | --- | --- | --- | --- | --- |
| *LCORL* | exon1-4 | CAGAGGGAAGGTAGTGACACG | GGTAACTTTCTGCTTGGCATTC | 550 | 61 | cLCORL_1 |
| *LCORL* | exon1-3 | AGCAGTATGGCTCGCTTGAG | CCAGTCAGTCAGCTCTTCTGG | 547 | 63 | cLCORL_2 |
| *LCORL* | exon3-7.1 | CCAGAAGAGCTGACTGACTGG | TTTGAGGTATGCCATAAAGTATGC | 688 | 61 | cLCORL_3 |
| *LCORL* | exon4-7.1 | AGATTGAATGCCAAGCAGAAAG | GAGGTAACTGGAAGCTGTTGG | 767 | 61 | cLCORL_4 |
| *LCORL* | exon6-7.1 | GTCATCCATATGCGATCCTTC | GAGGTAACTGGAAGCTGTTGG | 412 | 61 | cLCORL_5 |
| *LCORL* | exon3-7.2 | TGAACCAGAAGAGCTGACTGAC | TCCCATCAGACAAATTCAGTTTC | 599 | 60 | cLCORL_6 |
| *LCORL* | exon5-7.2 | CTCCCTTCAGGAAGAACAGG | TCAAACTGGGGACAATTACG | 631 | 60 | cLCORL_7 |
| *LCORL* | exon2 | CTCTGTGTATATTGACACCTCAAAG | TTTCTAATTAAACAAGATCACAAATTC | 343 | 57 | gLCORL_1 |
| *LCORL* | exon3 | AACCATTGCTGTGGAGAGTG | CCACTGTAAGTCACAAACTTGC | 271 | 59 | gLCORL_2 |
| *LCORL* | exon4 | TTTAAAAGGTAGAACAAATGAGC | AACGTGTGTGTGAGAAAAATTC | 315 | 57 | gLCORL_3 |
| *LCORL* | exon5 | TGCTAAGTCCCACAAACCAC | AAAGAGGCTGAATTACTCTCAAAG | 592 | 58 | gLCORL_4 |
| *LCORL* | exon6 | TTTAGGTCATGAATTAGTAGATCAGC | TGAAAGGTATGAGAGACACACAC | 341 | 57 | gLCORL_5 |
| *LCORL* | exon7.1 | GCGTTTGCTTTGTTAAGGAG | ACCATCAGGTTGTGGTTTTG | 680 | 58 | gLCORL_6.1A |

**Table S4 continued.**

| Gene | Gene region | Forward primer (5’-3’) | Reverse primer (5’-3’) | Product size (bp) | AT^a^ (°C) | Name |
| --- | --- | --- | --- | --- | --- | --- |
| *LCORL* | exon7.1 | TCCCAACAGCTTCCAGTTAC | TCTCCTGTCAGAGTGAGCATC | 829 | 58 | gLCORL_6.1B |
| *LCORL* | exon7.2 | ATCAAGCAAGGAGGCAGAAG | TGCAAAATTCCACAATGGTC | 550 | 59 | gLCORL_6.2 |
