# Supplementary material for: Expression Levels of LCORL Are Associated with Body Size in Horses
Source: PLoS One. 2013 Feb 13;8(2):e56497. doi: 10.1371/journal.pone.0056497 (PMC3572084; doi:10.1371/journal.pone.0056497)
Supplement: Table S5 — Samples used for expression analysis of LCORL , NCAPG and DCAF16 . The breed, height at the withers, genotype, sex, age at the time of sampling and the time of sampling are shown. (DOC) [file pone.0056497.s008.doc]

**Table S5**. **Samples used for expression analysis of *LCORL, NCAPG* and *DCAF16*.** The breed, height at the withers, genotype, sex, age at the time of sampling and the time of sampling is shown.

| Breed | Height at the withers  (cm) | Genotype  (BIEC2-808543) | Sex  (1=male, 2=female) | Age  (in years, at the time of sampling) | Time of sampling |
| --- | --- | --- | --- | --- | --- |
| Arabian | 150 | T/T | 1 | 3 | AM* |
| Arabian | 155 | T/T | 1 | 3 | AM* |
| Arabian | 148 | T/T | 1 | 4 | AM* |
| Arabian | 146 | T/T | 1 | 9 | AM* |
| Arabian | 157 | T/T | 1 | 6 | AM* |
| Arabian | 148 | T/T | 2 | 10 | AM* |
| Arabian | 140 | T/T | 2 | 2 | AM* |
| Arabian | 145 | T/T | 2 | 6 | AM* |
| Arabian | 148 | T/T | 2 | 3 | AM* |
| Arabian | 142 | T/T | 2 | 8 | AM* |
| Arabian | 150 | T/T | 2 | 5 | AM* |
| Hanoverian | 166 | T/T | 1 | 5 | AM* |
| Hanoverian | 162 | T/T | 1 | 6 | AM* |
| Hanoverian | 162 | T/T | 1 | 6 | AM* |
| Hanoverian | 165 | T/T | 1 | 5 | AM* |
| Hanoverian | 168 | C/T | 1 | 3 | AM* |
| Hanoverian | 168 | C/T | 2 | 6 | AM* |
| Hanoverian | 167 | C/T | 2 | 6 | AM* |

*AM=ante meridiem

**Table S5. continued.**

| Breed | Height at the withers (cm) | Genotype  (BIEC2-808543) | Sex  (1=male, 2=female) | Age  (in years, at the time of sampling) | Time of sampling |
| --- | --- | --- | --- | --- | --- |
| Hanoverian | 170 | C/C | 1 | 3 | AM* |
| Hanoverian | 172 | C/C | 1 | 6 | AM* |
| Hanoverian | 169 | C/C | 1 | 6 | AM* |
| Hanoverian | 170 | C/C | 1 | 7 | AM* |
| Hanoverian | 175 | C/C | 1 | 8 | AM* |
| Hanoverian | 172 | C/C | 2 | 3 | AM* |
| Welsh Section A | 120 | T/T | 1 | 9 | AM* |
| Welsh Section A | 115 | T/T | 1 | 8 | AM* |
| Welsh Section A | 118 | T/T | 1 | 6 | AM* |
| Dülmener | 126 | T/T | 1 | 5 | AM* |
| Dülmener | 133 | T/T | 1 | 6 | AM* |
| Dülmener | 123 | T/T | 2 | 8 | AM* |
| Dülmener | 130 | T/T | 2 | 6 | AM* |
| Dülmener | 118 | T/T | 2 | 8 | AM* |
| Dülmener | 133 | T/T | 2 | 4 | AM* |
| Dülmener | 131 | T/T | 2 | 1 | AM* |
| Dülmener | 135 | T/T | 2 | 7 | AM* |
| Dülmener | 138 | C/T | 1 | 1 | AM* |
| Dülmener | 134 | C/T | 1 | 5 | AM* |
| Dülmener | 136 | C/T | 2 | 4 | AM* |
| Dülmener | 138 | C/T | 2 | 4 | AM* |

*AM=ante meridiem

**Table S5. continued.**

| Breed | Height at the withers (cm) | Genotype  (BIEC2-808543) | Sex  (1=male, 2=female) | Age  (in years, at the time of sampling) | Time of sampling |
| --- | --- | --- | --- | --- | --- |
| Rhenish German Draught | 168 | C/C | 1 | 3 | AM* |
| Rhenish German Draught | 170 | C/C | 1 | 2 | AM* |
| Rhenish German Draught | 170 | C/C | 1 | 7 | AM* |
| Rhenish German Draught | 169 | C/C | 1 | 1 | AM* |
| Rhenish German Draught | 158 | C/C | 2 | 7 | AM* |
| Rhenish German Draught | 169 | C/C | 2 | 6 | AM* |
| Rhenish German Draught | 169 | C/C | 2 | 2 | AM* |
| Rhenish German Draught | 174 | C/C | 2 | 4 | AM* |

*AM=ante meridiem
