# Supplementary material for: Expression Levels of LCORL Are Associated with Body Size in Horses
Source: PLoS One. 2013 Feb 13;8(2):e56497. doi: 10.1371/journal.pone.0056497 (PMC3572084; doi:10.1371/journal.pone.0056497)
Supplement: Table S6 — Primer sequences, their product sizes, annealing temperatures (AT) and TaqMan probes used for real-time quantitative PCR (RT-qPCR) for LCORL , NCAPG and DCAF16 using GAPDH as reference gene. (DOC) [file pone.0056497.s009.doc]

**Table S6.** **Primer sequences, their product sizes, annealing temperatures (AT) and TaqMan probes used for real-time quantitative PCR (RT-qPCR) for *LCORL, NCAPG and DCAF16* using *GAPDH* as reference gene.**

| Gene | Forward primer (5’-3’) | Reverse primer (5’-3’) | Product size (bp) | AT^a^ (°C) | TaqMan probe |
| --- | --- | --- | --- | --- | --- |
| *LCORL* | CTTTATGGACCACGGCTACGA | TTTTCATCCATAGACCAGTCAGTCA | 86 | 60 | TATTTGAAGACTGTGAACCAGA |
| *NCAPG* | CGACTTCAGGATCCCAAAGATG | CACTGCCCGCCTAACTTCTG | 96 | 60 | CAGTGGTTAACGCATATG |
| *DCAF16* | GCACTGCAATTTGCCACCAA | AGCAACAACCAGACACACATGAATTA | 106 | 60 | TCCCATACCTGAATACCT |
| *GAPDH* | GGCTGCTTTTAACTCTGGCAAA | GCCTTGACTGTGCCATGGA | 126 | 60 | ACATGGTCTACATGTTTCAGT |
